# A study on the impact of project-based learning on students’ learning motivation in animation programs

**DOI:** 10.3389/fpsyg.2025.1722170

**Published:** 2026-01-02

**Authors:** Qiuwan Zhang, Chang Lin

**Affiliations:** 1 Guangzhou Academy of Fine Arts, School of Visual Arts Design, Guangzhou, China; 2 The Innovation School of Great Bay Area, Guangzhou Academy of Fine Arts, Guangzhou, China

**Keywords:** animation teaching, project-based learning, learning motivation, curriculum reform, animation majors

## Abstract

Project-based learning (PBL) is widely used in animation education, yet empirical evidence on how specific PBL dimensions relate to students’ learning motivation remains limited. Drawing on a survey of 319 animation majors and recent graduates in China, this study examines students’ perceptions of PBL implementation and their learning motivation using a PBL scale and a learning motivation scale. Correlation and hierarchical regression analyses were conducted to test the predictive effects of PBL on multiple motivation dimensions. The results show a significant positive relationship between students’ perceived PBL experiences and learning motivation. Specifically, problem-driven learning exerted the strongest effects on challenge, enthusiasm, reliance on others’ evaluation, preference for simple tasks, and focus on interpersonal competition; project design significantly predicted preference for simple tasks and interpersonal competition; and project implementation positively influenced enthusiasm, whereas project evaluation showed no significant predictive effect. These findings deepen understanding of the motivational mechanisms associated with PBL in animation programs and offer practical implications for improving PBL instructional effectiveness.

## Introduction

At the end of the 20th century, with the Chinese government’s increasing attention and support for the animation industry, the animation programs in universities across the country developed rapidly, showing a positive trend in the field of animation art education (Sun and Xiao, 2024). The “Development Plan for the New Generation of Artificial Intelligence” identifies the research and application of artificial intelligence technology as one of the key areas in the national scientific and technological layout. Additionally, the widespread use of artificial intelligence technology has unleashed social productivity and may have profound effects method of working, labor market structures and educational systems in various social domains (Damioli et al., 2021).

In the field of animation, the application of artificial intelligence technology is becoming increasingly widespread. For example, AI painting techniques can imitate and innovate different artistic styles, by learning from a large number of artistic works, thereby improving the efficiency and quality of painting (Liu and Zhu, 2025). Additionally, AI animation generation techniques utilize deep learning methods to automate the animations, reducing production cycles and costs (Wang et al., 2024). Although artificial intelligence technology to some extent may replaces manual labor, it also presents higher skill requirements for the animation industry.

Guangdong Province serves as a major center and hub for the animation industry in China, and the animation program is committed to nurturing a large number of excellent animation talents in the region. Besides, faced with the changing demands brought about by artificial intelligence technology, educators have increasingly emphasized educational approaches that promote classroom interaction and collaborative learning (Dai, 2024), such as flipped classrooms, blended learning, and PBL. What’s more, the flipped classroom teaching model transforms the learning of knowledge point into thematic learning (Shrimal, 2024) by having students preview course content before class and using class time for discussion, practice, and questioning, which enhances students’ learning enthusiasm (Bishop and Verleger, 2013). Researchers have constructed teaching models through action research (Chen and Jiang, 2014) to explore teaching and learning mode, approaches and strategies of flipped classrooms (Austin, 2025).

Meanwhile, blended learning combines online and offline teaching, providing diverse options for animation classroom instruction. Animation course teaching methods based on virtual reality technology are gradually attracted attention. By applying virtual reality technology to animation education, students can enhance their animation production skills and innovation abilities in an immersive learning environment (Deng et al., 2021), while also making full use of digital technology to provide personalized learning resources (Ji, 2021).

The origins of PBL can be traced back to 1918 when American educator William Heard Kilpatrick proposed the concept in his article titled “The Project Method: The Use of Purposeful Activity in the Education Process” (Howard, 2002). Correspondingly, he advocated that teaching activities should be centered around student needs. Furthermore, PBL refers to a teaching model where students explore complex, real-world problems and acquire knowledge and skills through activities such as solving problems, collaborations, researches, and presentations within the context of a project (Mergendoller et al., 2006). Thus, in the field of animation, students can engage in authentic animation creation processes, from needs analysis and concept design to production and presentation, comprehensively developing their animation skills and teamwork abilities.

Research on PBL in animation primarily focuses on implementation steps and practical case studies (Wang, 2024). Compared to traditional teaching methods, empirical research findings indicate that PBL has a positive impact on students’ academic achievements, and competencies (Purković and Prihoda, 2020; Zhang and Ma, 2023). For instance, students engaged in PBL demonstrate higher abilities in problem solving, collaboration, and communication (Blumenfeld et al., 1991). PBL also has a positive influence on learning motivation, as students exhibit more initiative and independent in PBL environments and show greater interest and engagement in their learning (Strobel and van Barneveld, 2009). Learning motivation is an important factor that affects learning outcomes. Currently, research on PBL in animation primarily revolves around exploring the integration of PBL with animation course content, while the relationship between PBL and learning motivation remains relatively limited in its investigation.

In conclusion, in order to thoroughly investigate the characteristics of PBL in the animation field and analyze the learning motivation of students and its impact, this study takes the PBL course in the animation programs as the research subject, aiming at provide an empirical research foundation for the innovation and exploration of animation teaching models. Based on the above objectives, the following research questions were formulated:

RQ1: How do students perceive and evaluate the implementation of project-based learning (PBL) in animation programs?

RQ2: What is the relationship between students’ perceptions of PBL and their learning motivation?

RQ3: Which specific dimensions of PBL (problem-driven, project design, project implementation, and project evaluation) have significant effects on students’ learning motivation?

## Research methodology

### Analytical framework

Constructivist learning theory underscores that students construct knowledge through solving real-world problems and teamwork. Learning is posited as an active, socially interactive process where students construct new knowledge through cooperation, exploration, and practice. Project-based learning (PBL) provides such an authentic and meaningful learning context, enabling learners to develop knowledge and skills through sustained inquiry, collaboration, and production of concrete outcomes. Drawing on the widely recognized PBL framework synthesized by Kokotsaki et al. (2016), which summarizes core characteristics of PBL such as authenticity, student agency, collaboration, iterative feedback, and creation of a final product, this study adopts a four-dimension analytical structure—problem-driven inquiry, project design, project implementation, and project evaluation. This structure aligns with mainstream PBL conceptualizations (Bell, 2010) and fits the workflow of animation production courses, allowing for systematic examination of how specific dimensions of PBL relate to students’ learning motivation.

Problem-driven serves as the starting point for PBL, highlighting the goal of learning as solving real-life problems. By situating learning within real-world contexts, project questions stimulate students’ interest and curiosity, fostering active engagement in the learning process. Project design refers to the process of designing and planning learning activities in PBL, which involves setting learning objectives, choosing project themes, formulating learning tasks and activities, and organizing student collaboration and interaction. The crux of project design is to align learning activities with educational objectives and problem-driven approaches to ensure that students gain knowledge and improve skills in practice. Project implementation denotes the process by which students carry out actual projects under teacher guidance. Through exploration, practice, and collaboration, students apply their knowledge and skills to solve problems, gaining practical experience. Project evaluation is the assessment of student performance and outcomes within PBL. What’s more, beyond academic performance, project evaluation also focuses on students’ thought processes, strategies of solving problem, and the quality of teamwork to aid students in recognizing their learning achievements and areas for improvement, providing guidance for future learning endeavors.

### Data collection

This study selected sophomores to seniors of animation programs as research subjects, employing random and stratified sampling methods, resulting in 319 valid samples. Additionally, interviews with the heads of the Animation Department revealed that freshmen had not yet engaged in PBL related courses and were thus excluded from the study. The questionnaire survey was conducted between August 12 and August 26, 2023, in Guangdong Province. Data were collected via Wenjuanxing online platform and participation was voluntary and anonymous. Prior to distribution, consent was obtained from all participants, and data were screened for completeness before analysis.

### Research tools

**Figure 1:**
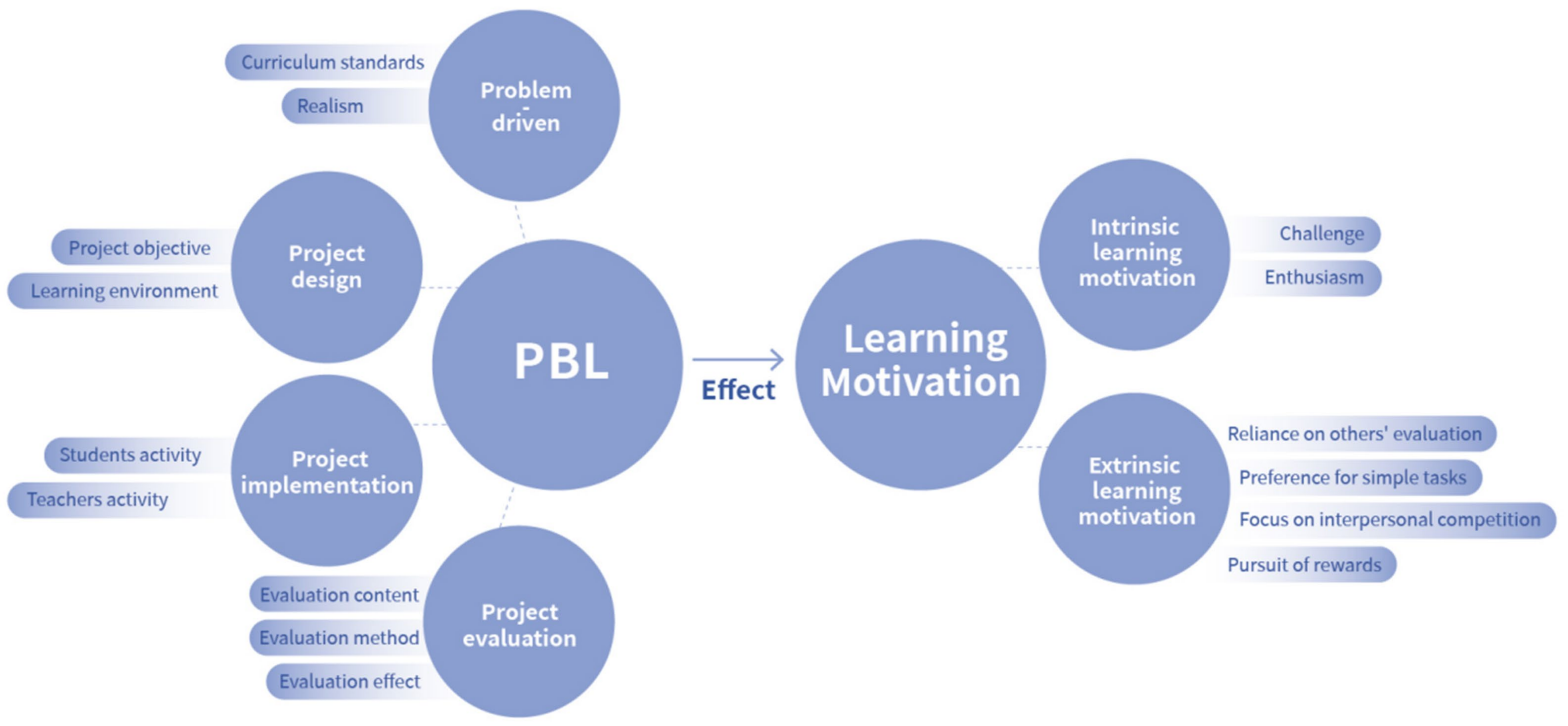
PBL and learning motivation questionnaire framework.

The study investigated PBL and learning motivation via questionnaires. The PBL questionnaire was developed based on the PBL process and in reference to the “PBL Evaluation Indicators” (Pan et al., 2021). Adjusted to suit the cognitive status of students and expert advice, the final “Animation program PBL scale” was created. This evaluation based on curriculum restructuring for PBL, highlights distinctive features in the teaching of animation, where subject knowledge is integrated with specific projects, transforming subject knowledge into PBL activities, aligning well with this study. Furthermore, the questionnaire is a multidimensional measurement tool, divided into four dimensions: problem-driven, project design, project implementation, and project evaluation, comprising 25 items. The research framework is illustrated in Figure 1.

To establish a solid theoretical foundation for examining students’ learning motivation, this study adopts Self Determination Theory. Early work by Deci (1972) first distinguished between intrinsically motivated behaviors, which originate from personal interest or satisfaction, and extrinsically motivated behaviors that are driven by external incentives. This theoretical perspective was further expanded by Deci and Ryan (1985), who systematically explained how social and contextual factors influence the internalization of motivation. The most comprehensive articulation of the theory was provided by Ryan and Deci (2000), who emphasized that intrinsic motivation reflects a natural human tendency to learn and explore, while extrinsic motivation varies in the degree to which it is autonomous rather than controlled. According to their framework, the quality of both intrinsic and extrinsic motivation depends on the fulfillment of three basic psychological needs: autonomy, competence, and relatedness, which directly influence learners’ engagement, persistence, and performance. The clarity, developmental grounding, and strong empirical support for Self-Determination Theory make it especially well suited for educational research, particularly when examining how students participate in structured, collaborative project-based activities.

Based on this theoretical foundation, the measurement of learning motivation in this study draws from the Work Preference Inventory developed by Amabile et al. (1994). Although originally designed to assess motivational orientations in work settings, the Work Preference Inventory has been widely applied in educational contexts because its core constructs of intrinsic and extrinsic motivation align closely with students’ academic behaviors, learning engagement and task-selection preferences. Learners’ tendencies toward challenge seeking, curiosity-driven behavior and sensitivity to external evaluation are conceptually consistent with the motivational patterns identified in academic learning research. Therefore, the framework of the Work Preference Inventory provides an appropriate basis for adapting motivation measures in the context of animation education.

In recent years, educational researchers have further extended the application of intrinsic and extrinsic motivational frameworks in project-based learning environments. A recent meta-analysis by Wijnia et al. (2024) demonstrated that project-based, problem-based and case-based learning structures significantly influence both intrinsic and extrinsic forms of motivation across diverse educational settings. Their findings support the validity of applying intrinsic–extrinsic motivation frameworks to studies that examine instructional models emphasizing authenticity, student autonomy and collaborative inquiry.

Guided by these theoretical and empirical foundations, this study employs an adapted motivation scale that includes two intrinsic dimensions, challenge and enthusiasm, and four extrinsic dimensions, reliance on external evaluation, preference for simple tasks, interpersonal competition and reward pursuit, with a total of 30 items.

The survey questionnaires were structured using a five-point Likert scale, where 1 signified “strongly disagree” and 5 represented “strongly agree.” Higher scores in the various dimensions of the PBL evaluation indicated a higher student appraisal for that dimension. Similarly, higher scores in the dimensions of learning motivation denoted a higher level of that dimension. All items were randomly distributed in the questionnaire, with reverse scoring items included. A total of 335 questionnaires were distributed, of which 319 were valid, resulting in a 95.22% validity rate.

The Cronbach’s Alpha for the PBL and learning motivation scales were 0.985 and 0.957, respectively, with the dimensions of problem-driven, project design, project implementation, project evaluation, intrinsic learning motivation, and extrinsic learning motivation yielding scores of 0.905, 0.934, 0.964, 0.963, 0.951, and 0.914, which all above 0.900. This indicated high internal consistency for both scales, with highly ideal reliability indices, making the scales reliable tools for measuring PBL.

After passing the reliability test, the questionnaires were subjected to test validity. The suitability of factor analysis was examined using the Bartlett test method and measured with the Kaiser-Meyer-Olkin (KMO) test. The closer the KMO value is to 1, the more suitable it is for factor analysis (Wu, 2010). The KMO values for the PBL scale, learning motivation scale, and their respective dimensions were all greater than 0.80. The KMO value for the scale of PBL was 0.972, with the problem-driven, project design, project implementation, and project evaluation dimensions having KMO values of 0.822, 0.910, 0.957, and 0.949, respectively. The KMO value for the scale of learning motivation was 0.952, with intrinsic and extrinsic learning motivation at 0.940 and 0.937, respectively. Bartlett’s test of sphericity yielded significant results at the 0.000 for all variables, indicating the presence of common factors among them and their suitability for factor analysis.

### Data analysis

The study utilized SPSS 26.0 software to perform descriptive statistical analysis on the data from the PBL evaluation and learning motivation questionnaires. Correlation analysis and hierarchical regression analysis were conducted to investigate the impact of PBL on students’ learning motivation.

### Research findings

#### Descriptive statistical analysis

##### Characteristics of survey participants

The study analyzed the demographic characteristics of the participants, which were 86 males (26.96%) and 233 females (73.04%). Regarding their origin, 260 (81.50%) came from urban areas, while 59 (18.50%) were from rural areas. In terms of grade, there were 85 sophomores (26.65%), 83 juniors (26.02%), 101 seniors (31.66%), and 50 graduates (15.67%). To obtain more comprehensive and authentic data on PBL, this study included graduates as research subjects. The rationale behind this decision is that the graduation creations in the Animation program are predominantly conducted in a PBL format, and graduates can provide more candid and uninhibited feedback on this educational approach.

##### Students’ PBL and learning motivation status

The research data indicated that students’ overall evaluation of PBL was quite high, with an average level of 3.7103. The scores across all dimensions were relatively high (*M* > 3.60), suggesting that the aspects of problem-driven, project design, project implementation, and project evaluation were generally affirmed by the students. The study investigated students’ learning in terms of challenge, enthusiasm, reliance on others’ evaluation, preference for simple tasks, focus on interpersonal competition, and pursuit of rewards, as shown in Table 1. The overall average level of students’ learning motivation was 3.7679, which is relatively high. The average scores from high to low were enthusiasm, challenge, preference for simple tasks, focus on interpersonal competition, pursuit of rewards, and reliance on others’ evaluation. This indicates that students’ intrinsic motivation for learning (*M* = 3.9299) was overall higher than their extrinsic motivation (*M* = 3.6262), with enthusiasm scoring the highest average, suggesting that students have a high level of enthusiasm for PBL.

**Table 1:** Descriptive statistics of PBL and students’ learning motivation.

Variable	*N*	Min	Max	*M*	SD
Problem-driven	319	1	5	3.7351	0.84068
Project design	319	1	5	3.6829	0.8839
Project implementation	319	1	5	3.7492	0.89827
Project evaluation	319	1	5	3.6846	0.92298
Challenge	319	1	5	3.8452	0.7798
Enthusiasm	319	1	5	4.0428	0.78045
Reliance on others’ evaluation	319	1	5	3.4347	0.65703
Preference for simple tasks	319	1	5	3.7868	0.82083
Focus on interpersonal competition	319	1	5	3.7359	0.86022
Pursuit of rewards	319	1	5	3.6599	0.91088
PBL scale	319	1	5	3.7103	0.86216
Learning motivation scale	319	1	5	3.7679	0.66275

*M*, mean; SD, standard deviation; Min, Minimum; Max, Maximum.

### Correlation analysis

The results of the correlation analysis between PBL and students’ learning motivation are presented in Table 2. There was a moderate positive correlation between each dimension of PBL and each dimension of learning motivation (0.40 ≤ *r* ≤ 0.70, *p* < 0.01). The correlation of PBL with reliance on others’ evaluation and pursuit of rewards was slightly lower than with challenge, enthusiasm, preference for simple tasks, and focus on interpersonal competition. There remains potential for further analysis among these variables.

**Table 2:** Correlation analysis of each dimension (*N* = 319).

Dimension	P	PD	PI	PE	C	E	ROOE	PFST	FOIC	POR
P	1									
PD	0.889**	1								
PI	0.874**	0.913**	1							
PE	0.877**	0.911**	0.932**	1						
C	0.675**	0.676**	0.677**	0.663**	1					
E	0.655**	0.623**	0.633**	0.615**	0.796**	1				
ROOE	0.544**	0.538**	0.507**	0.519**	0.598**	0.571**	1			
PFST	0.634**	0.633**	0.611**	0.615**	0.574**	0.695**	0.718**	1		
FOIC	0.635**	0.635**	0.611**	0.611**	0.732**	0.617**	0.723**	0.677**	1	
POR	0.494**	0.501**	0.468**	0.489**	0.555**	0.533**	0.622**	0.628**	0.644**	1

**p < 0.01.

P, problem-driven; PD, project design; PI, project implementation; PE, project evaluation.

C, challenge; E, enthusiasm; ROOE, reliance on others’ evaluation; PFST, preference for simple tasks; FOIC, focus on interpersonal competition; POR, pursuit of rewards.

### Hierarchical regression analysis

To examine how project-based learning predicts students’ learning motivation, hierarchical multiple regression analyses were conducted. In all models, demographic variables were entered in the first step, followed by the four PBL predictors in the second step. The general regression equation for Step 2 of each model was:


$$\begin{array}{l} Y=\beta 0+\beta ₁(\text{Gender})+\beta ₂(\text{Place of Origin})+\beta ₃(\text{Grade})+\\ \beta ₄(\text{Problem Driven})+\beta 5(\text{Project Design})+\\ \beta 6(\begin{array}{l} \text{Project}\;\\ \text{Implementation} \end{array})+\beta 7(\begin{array}{l} \text{Project}\;\\ \text{Evaluation} \end{array})+\varepsilon \end{array}$$

where *Y* represents one of the six motivation dimensions: challenge, enthusiasm, reliance on others’ evaluation, preference for simple tasks, focus on interpersonal competition or pursuit of rewards. The purpose of the hierarchical analysis was to determine whether PBL variables contributed significant additional variance in motivation beyond demographic factors. The null hypothesis for each model stated that PBL predictors would not significantly predict the motivational outcome, while the alternative hypothesis stated that at least one PBL predictor would be significant.

Across all models, the control variables alone did not significantly predict motivation in Step 1, with *R*^2^ values between 0.001 and 0.013. When the PBL variables were added, all models became statistically significant, indicating meaningful explanatory power from PBL characteristics.

For challenge, the full model was significant, *R*^2^ = 0.503, *F*(7, 311) = 44.933, *p* < 0.001, with a large improvement in variance explained over Step 1 (Δ*R*^2^ = 0.501). Problem-driven learning was the only significant predictor, *β* = 0.508, *p* < 0.01, indicating that clearly structured and meaningful problems strongly enhance students’ willingness to take on challenging tasks.

For enthusiasm, the full model was significant, *R*^2^ = 0.452, *F*(7, 311) = 36.604, *p* < 0.001, with Δ*R*^2^ = 0.446. Both problem-driven learning (*β* = 0.569, *p* < 0.001) and project implementation (*β* = 0.206, *p* < 0.05) significantly predicted enthusiasm. This suggests that the formulation of project problems and the hands-on implementation process both play important roles in stimulating students’ emotional involvement in animation learning.

For reliance on others’ evaluation, the full model was significant, *R*^2^ = 0.315, *F*(7, 311) = 20.441, *p* < 0.001, with Δ*R*^2^ = 0.314. Problem-driven learning was the only significant predictor, *β* = 0.361, *p* < 0.01, implying that problem-oriented activities encourage students to attend more closely to external feedback and peer judgment.

For preference for simple tasks, the full model was significant, *R*^2^ = 0.442, *F*(7, 311) = 35.197, *p* < 0.001, with Δ*R*^2^ = 0.431. Both problem-driven learning (*β* = 0.277, *p* < 0.01) and project design (*β* = 0.171, *p* < 0.05) were significant predictors. When projects involve complex collaboration or competitive elements, students may strategically select less demanding tasks to manage workload and group expectations.

For focus on interpersonal competition, the full model was significant, *R*^2^ = 0.429, *F*(7, 311) = 33.436, *p* < 0.001, with Δ*R*^2^ = 0.423. Problem-driven learning (*β* = 0.312, *p* < 0.01) and project design (*β* = 0.183, *p* < 0.05) significantly predicted competitive focus, suggesting that project visibility, performance comparison and design structure heighten students’ awareness of peer competition.

Finally, the model predicting pursuit of rewards was also significant, *R*^2^ = 0.293, *F*(7, 311) = 18.375, *p* < 0.001, with Δ*R*^2^ = 0.279, although none of the individual PBL variables were significant predictors. This indicates that although the PBL context as a whole relates to reward-oriented tendencies, specific PBL characteristics do not directly drive students’ pursuit of external rewards.

Overall, the findings show that problem-driven learning is the most consistent and influential predictor across multiple motivational dimensions, particularly those relating to intrinsic motivation. Project design and project implementation demonstrate more targeted effects, while project evaluation does not significantly predict motivation. These results underscore the importance of core PBL features in shaping motivation within animation education settings.

In summary, after controlling for demographic variables, the findings indicate that PBL meaningfully contributes to students’ learning motivation. Among the four dimensions of PBL, the problem-driven component emerged as the most influential predictor, significantly enhancing students’ sense of challenge, enthusiasm, reliance on others’ evaluation, preference for simple tasks, and focus on interpersonal competition. Project design also showed significant positive effects on students’ preference for simple tasks and competitive orientation, while project implementation significantly promoted learning enthusiasm. In contrast, project evaluation did not exert a significant predictive effect on any motivational dimension. In addition, the standardized regression coefficients revealed stronger effects on intrinsic motivation than on extrinsic motivation, suggesting that PBL is particularly effective in fostering students’ interest-driven and self-sustaining engagement in animation learning (see Table 3).

**Table 3:** Regression analysis on the influence of PBL on learning motivation.

Variable	C		E		ROOE		PFST		FOIC		POR	
	Model 1	Model 2	Model 1	Model 2	Model 1	Model 2	Model 1	Model 2	Model 1	Model 2	Model 1	Model 2
(Constant)	32.266	10.189	24.36	8.708	21.36	10.303	14.908	4.082	16.337	5.084	7.388	2.61
Gender	−0.613	−0.741	0.179	0.067	−0.209	−0.214	0.039	0.009	−0.184	−0.213	−0.351	−0.351
Place of origin	−0.398	0.341	0.528	1.005	−0.25	0.027	0.718	1.049**	−0.141	0.199	0.263	0.392
Grade	0.008	0.501*	−0.302	0.035	−0.027	0.242	−0.197	0.054	−0.264	−0.004	0.066	0.186*
P	—	0.508**		0.569***		0.361**		0.277**		0.312**		0.108
PD	—	0.224		0.056		0.184		0.171*		0.183*		0.093
PI	—	0.243		0.206*		−0.053		0.036		0.034		−0.027
PE	—	0.027		−0.035		0.059		0.026		0.014		0.043
*R*^2^	0.002	0.503	0.006	0.452	0.001	0.315	0.011	0.442	0.007	0.429	0.013	0.293
∆*R*^2^	0.002	0.501	0.006	0.446	0.001	0.314	0.011	0.431	0.007	0.423	0.013	0.279
*F*	0.224	44.933***	0.653	36.604***	0.105	20.441***	1.151	35.197***	0.713	33.436***	1.429	18.375***

*p < 0.05, **p < 0.01, ***p < 0.001.

P, problem-driven; PD, project design; PI, project implementation; PE, project evaluation.

C, challenge; E, enthusiasm; ROOE, reliance on others’ evaluation; PFST, preference for simple tasks; FOIC, focus on interpersonal competition; POR, pursuit of rewards.

## Discussion

**Figure 2:**
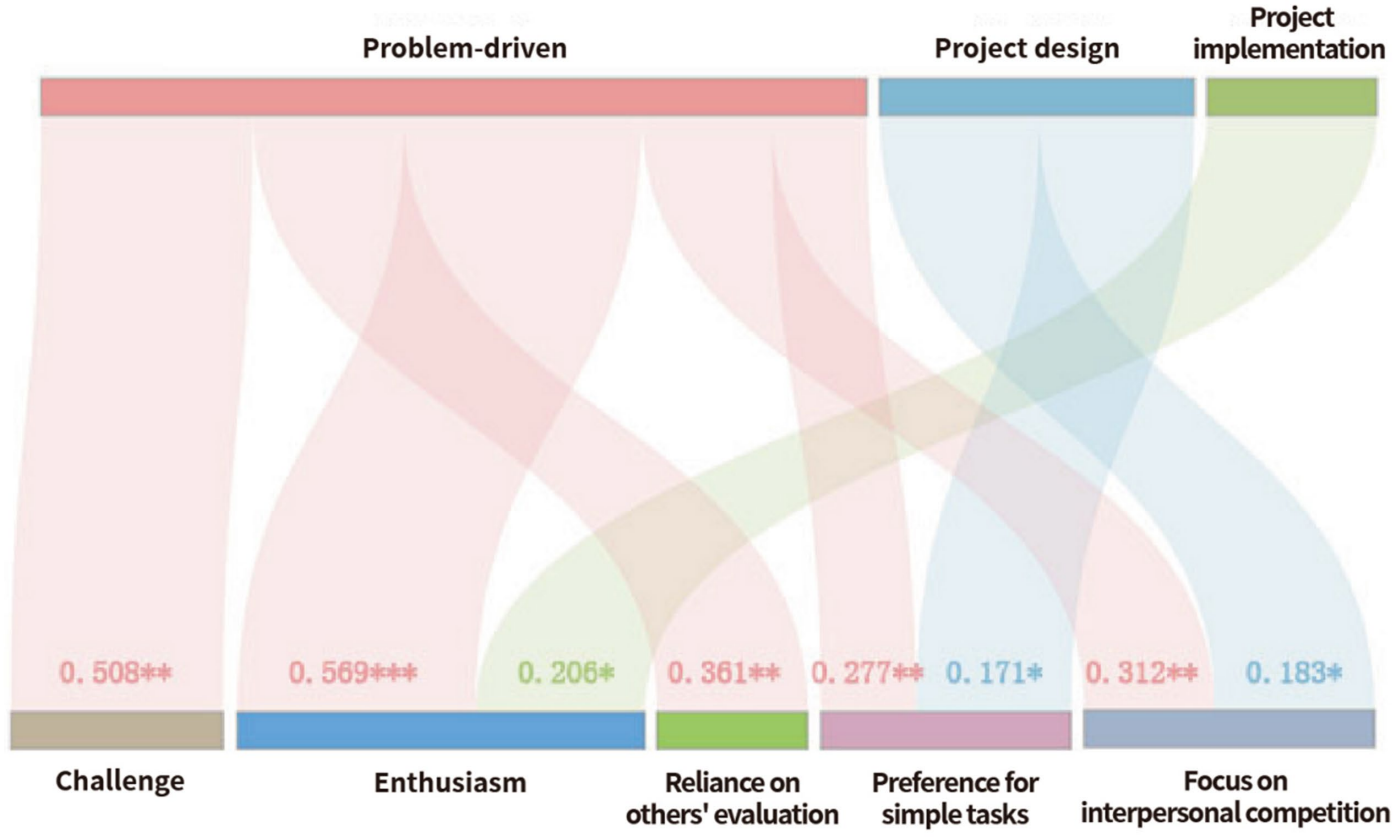
The relationship between PBL and learning motivation.

This study empirically explored the impact of PBL on students’ learning motivation, addressing RQ1–RQ3. The results indicate that animation students generally hold positive perceptions of PBL implementation and demonstrate strong learning motivation, with intrinsic motivation significantly higher than extrinsic motivation. The influence of PBL on motivation appears across multiple dimensions, as shown in Figure 2. Hence, in the teaching process of PBL, teachers should scientifically design projects that align with students’ learning characteristics and motivational patterns. Although students generally reported positive evaluations of PBL, these descriptive scores were not used to infer that PBL directly increased their learning motivation. The connection between PBL and motivation in this study was established through hierarchical regression modeling, in which the four PBL dimensions served as independent variables and the six motivation dimensions were dependent variables. This analytical design allows us to examine whether variation in students’ experiences statistically predicts variation in their learning motivation after controlling for demographic factors. Thus, the influence of PBL on motivation was not derived from students’ subjective ratings alone but was demonstrated through statistically significant predictive relationships identified in the regression models. With this methodological clarification in place, it becomes meaningful to interpret how specific PBL features shape students’ motivational responses.

Firstly, problem-driven dimension of PBL exerts a strong positive influence on students’ sense of challenge, enthusiasm, reliance on others’ evaluation, preference for simple tasks, and focus on interpersonal competition. The regression results show that the effect of problem-driven learning on intrinsic motivation is significantly stronger than on extrinsic motivation. Designing animation projects with high authenticity and compelling content can effectively stimulate students’ intrinsic learning motivation, sparking intense enthusiasm for the problem and a strong desire to explore solutions. This aligns with international research findings that rich and authentic problem design effectively stimulates students’ enthusiasm for learning and spirit of exploration (Savin-Baden, 2000). Moreover, problem-driven also impacts extrinsic learning motivation, such as gaining appreciation from others through project results, or choosing simple tasks to satisfy a sense of self-worth. However, the influence of problem-driven on intrinsic motivation is significantly greater than on extrinsic motivation (Wellhöfer and Lühken, 2022), indicating it more readily inspires students’ internal interest in learning and urgency. In the design of project problems, teachers should consider the compatibility with course standards and place greater emphasis on choosing projects with strong realism and appeal, as this is more conducive to stimulating active student participation.

Secondly, project design significantly affects students’ preference for simple tasks and their focus on interpersonal competition. Effective project design should center around clear learning objectives aligned with course standards, and these objectives should be attainable given the students’ abilities. Additionally, project design should provide ample instructional resources, such as video tutorials and course materials, to meet the needs of students’ active learning. Sufficient educational resources can reduce students’ cognitive load, allowing them the option to choose simple learning tasks and thus satisfy their expectations of competing with peers upon task completions. The design of appropriate difficulty and the provision of external rewards will influence learners’ motivational choices (Clay et al., 2022). This result is consistent with more recent findings that transparent design structures and incremental feedback significantly strengthen students’ sense of self-efficacy and sustained engagement in project-based courses (Krsmanovic, 2021). Therefore, while establishing clear learning objectives, project design should give adequate support for students to build a learning environment that balances difficulty and external competitive opportunities.

Thirdly, project implementation significantly and positively inspires students’ enthusiasm for learning. Project implementation mainly assesses students’ learning activities and teachers’ instructional activities. Active participation in animation-based PBL enables students to apply theoretical knowledge in practice, develop production skills, and gain experiential understanding through peer and teacher collaboration. Such interactions directly influence learning experiences and, consequently, students’ enthusiasm and effectiveness. This finding is consistent with recent research showing that structured team collaboration and timely formative feedback can significantly enhance learners’ engagement and creative outcomes in project-based settings (Raymundo, 2020). Therefore, educators should pay attention to students’ states in the course of project implementation, guide their exploration independently, and provide feedback and guidance as necessary. Also, fostering students’ team collaboration awareness, guiding teams to cooperate based on individual strengths, and creating an atmosphere of mutual trust and support are essential. Additionally, the teacher’s ability to manage project progress can impact the outcome, as coordinating resource allocations among teams will benefit presentation of the final product.

What’s more, project evaluation does not significantly impact students’ learning motivation. Project evaluation encompasses aspects of content, methods and evaluation. Typically, evaluation model combining teacher assessment, peer evaluation, and self—assessment is used to comprehensively assess students’ performance at various stages and final outcomes (Chanpet et al., 2020). The findings illustrate that animation students have a high level of intrinsic motivation, focusing more on learning interest and experience, with external evaluations having less impact (Bülbül and Kuzu, 2021). This may partly interpret why project evaluation does not significantly influence learning motivation. This result aligns with other studies suggesting that different evaluation models do not significantly differentiate between high and low student motivation levels (Cress and Kimmerle, 2008). As the animation field attaches great importance to creativity cultivation, visual design, and aesthetic education, employing diversified and formative evaluation methods is appropriate. Therefore, educators should, in line with the characteristics of the animation discipline, focus on evaluating students’ initial design, stage drawings, and process management skills; assess their practical skills in teamwork and software usage for completing projects (Gabrijelčič Tomc, 2020); and motivate students to establish personal characteristics in narrative, visual representation, and experimental innovation. Recent research in arts and design education has also emphasized that multi-dimensional assessment systems, combining process evaluation, peer review, and self-reflection, can significantly enhance students’ creative confidence and sustained motivation (Shao et al., 2024). Enhancing teaching quality through a comprehensive assessment of achievements at different stages is also recommended.

Nevertheless, the present study has certain limitations that should be acknowledged. The data were collected from animation majors in Guangdong Province, which may limit the generalizability of the findings to other regions or disciplines with different instructional cultures and learning environments. In addition, the cross-sectional research design restricts causal inference, as the observed associations between PBL dimensions and learning motivation cannot capture changes over time or reflect long-term developmental trajectories. Future research may adopt longitudinal or experimental designs and include more diverse samples to further strengthen the evidence base.

## Conclusion

In summary, this study demonstrated that the problem-driven, project design, and project implementation dimensions of PBL significantly enhance students’ learning motivation, particularly their intrinsic motivation. Furthermore, students’ intrinsic learning motivation is higher than extrinsic motivation. Additionally, demographic variables such as gender and grade showed only limited moderating effects. These findings suggest that well-structured, authentic, and collaborative PBL environments can effectively foster students’ enthusiasm and engagement in animation education. Educators can reflect on and improve their teaching methods based on these research findings to promote effective student learning. Nevertheless, the extensibility and universality of these results require further validation, and the mechanisms behind changes in learning motivation have not been deeply analyzed. Future studies could conduct more systematic investigations. Overall, this study provides a valuable empirical basis for refining PBL practice in creative education and encourages continued interdisciplinary dialogue on motivation and pedagogy.

## Data Availability

The original contributions presented in the study are included in the article/Supplementary material, further inquiries can be directed to the corresponding author.
