# Supplementary material for: A study on the impact of project-based learning on students’ learning motivation in animation programs
Source: Front Psychol. 2026 Jan 2;16:1722170. doi: 10.3389/fpsyg.2025.1722170 (PMC12808368; doi:10.3389/fpsyg.2025.1722170)
Supplement: Supplementary file 1 [file Data_Sheet_1.docx]

**Appendix**

See Tables 4, 5

**Table 4** Animation program PBL scale

| Primary Indicator | Secondary  Indicator | Number | Item |
| --- | --- | --- | --- |
| Problem-driven | Curriculum standards | B1 | The alignment between project problems and curriculum standards. |
|  |  | B2 | Project problems encompass key knowledge points of the subject units. |
|  | Realism | B3 | Project problems are open, challenging, and realistic issues. |
|  |  | B4 | Project problems are practical issues faced in real life. |
| Project design | Project objective | B5 | Project goals revolve around learning objectives related to animation discipline knowledge and content design. |
|  |  | B6 | Project goals align with the cultivation objectives of students' animation abilities. |
|  |  | B7 | Project goals reflect the competency requirements of core qualities in animation majors. |
|  | Learning environment | B8 | Relevant teaching resources such as instructional videos and courseware are provided. |
|  |  | B9 | Adequate space, equipment, or facilities are available for project-based learning. |
|  |  | B10 | The project content is relevant to real situations |
| Project Implementation | Students activity | B11 | Students can raise challenging questions and seek guidance from teachers during the learning process. |
|  |  | B12 | Students can master technical methods within the project. |
|  |  | B13 | Effective student-teacher or student-student interactions occur, and students benefit from them. |
|  | Teachers activity | B14 | Teacher explanations or demonstrations on key and difficult points are clear. |
|  |  | B15 | Teacher explanations are enthusiastic or inspiring. |
|  |  | B16 | Teachers' abilities and expertise help students solve problems. |
|  |  | B17 | Teachers can effectively manage project progress, coordinate team collaboration, and make reasonable arrangements. |
| Project evaluation | Evaluation content | B18 | Classroom performance assessments can authentically reflect students' task completion (attitude, participation, group collaboration, etc.) from multiple perspectives. |
|  |  | B19 | Teacher feedback and evaluation are regularly integrated into project-based learning activities. |
|  |  | B20 | The evaluation criteria for learning tasks provided by teachers are specific, clear, and appropriate. |
|  |  | B21 | The final project work demonstrates students' mastery of the subject matter. |
|  |  | B22 | Students can provide timely feedback and suggestions on curriculum development. |
|  | Evaluation method | B23 | Evaluation methods are diverse, including not only teacher assessments but also peer evaluations, self-evaluations, and other forms. |
|  | Evaluation effect | B24 | Assignments or projects are valuable and assess the extent to which learning objectives are achieved. |
|  |  | B25 | Project evaluations ultimately reflect students' grasp of knowledge and skills. |

**Table 5** Learning motivation scale

| Primary Indicator | Secondary  Indicator | Number | Item |
| --- | --- | --- | --- |
| Intrinsic  learning motivation | Challenge | C1 | I enjoy independent thinking to solve difficulties. |
|  |  | C2 | I am very clear about the academic goals I need to achieve. |
|  |  | C3 | It is important for me to have the opportunity to express myself. |
|  |  | C4 | I hope that the work I engage in can provide me with opportunities to increase knowledge and skills. |
|  |  | C5 | I am more satisfied when I can set my own goals. |
|  | Enthusiasm | C6 | It is important for me to be able to do work that I enjoy. |
|  |  | C7 | I am willing to engage in work that interests me and allows me to focus and forget about everything else. |
|  |  | C8 | The most important thing for me is to love the work I do. |
|  |  | C9 | As long as I am doing something I enjoy, I don't care as much about grades and rewards. |
|  |  | C10 | Regardless of the outcome of what I do, as long as I feel that I have gained new experiences, I feel satisfied. |
|  |  | C11 | I do many things out of curiosity. |
|  |  | C12 | I don't care so much about what others think of my academic performance. |
|  |  | C13 | I care a lot about how others react to my opinions. |
|  |  | C14 | Being able to win the approval and appreciation of others is the main motivation that drives me to strive. |
| Extrinsic  learning motivation | Reliance on others' evaluation | C15 | I think it is meaningless to perform well at work if no one knows about it. |
|  |  | C16 | For me, the achievements I can win are the main motivation that drives me to strive. |
|  |  | C17 | I rarely think about grades and rewards. |
|  | Preference for simple tasks | C18 | I prefer relatively simple and straightforward tasks or assignments. |
|  |  | C19 | I enjoy doing work or tasks with clear procedural steps. |
|  |  | C20 | I prefer to choose work that I am confident in doing well, rather than work that requires me to give my all. |
|  |  | C21 | I prefer to have someone set clear goals for me in my work. |
|  | Focus on interpersonal competition | C22 | I want to know how well I can perform academically. |
|  |  | C23 | I hope that others will recognize my excellence in academics. |
|  |  | C24 | I am very clear that my goal or purpose is to pursue good grades. |
|  |  | C25 | For me, success means doing better than others. |
|  | Pursuit of rewards | C26 | Regardless of what I do, I always hope for some form of reward or compensation. |
|  |  | C27 | What concerns me more is not the work I do, but what I can gain from it. |
